# A Rare Cause of Chronic Cough: Intrathoracic Gossypiboma

**DOI:** 10.5812/iranjradiol.13933

**Published:** 2014-05-15

**Authors:** Dilber Yilmaz Durmaz, Behice Kaniye Yilmaz, Oya Yildiz, Yilmaz Bas

**Affiliations:** 1 Corum Chest Diseases Hospital, Corum, Turkey; 2 Department of Radiology, Corum Training and Research Hospital, Hitit University, Corum, Turkey; 3 Department of Thoracic Surgery, Corum Training and Research Hospital, Hitit University, Corum, Turkey; 4 Department of Pathology, Corum Training and Research Hospital, Hitit University, Corum, Turkey

**Keywords:** Thoracic Surgery, Cough, Chronic

## Abstract

Intrathoracic gossypiboma, a retained surgical sponge in the thoracic cavity, is a rare but serious complication of thoracic surgeries. A 70-year-old man presented with an eight-month history of cough. He had undergone coronary artery bypass surgery eight years ago. The posteroanterior chest X-ray revealed a well-marginated homogeneous opacity at the left hemithorax with striped appearance in the center. Thoracic CT revealed a pleural-based mass at the left lower lobe with a hyperdense rim. After the diagnosis of gossypiboma, it was removed surgically. Although rare after thoracic surgery, gossypibomas need to be considered in the differential diagnosis in case of respiratory symptoms.

## 1. Introduction

A gossypiboma or a textiloma is a non-absorbable surgical material with a cotton matrix, around which a foreign body reaction occurs (1). The term "gossypiboma" is bilingually derived from the Latin gossypium “cotton wool, cotton” and the Swahili-boma, (place of concealment), and describes a mass within a patient's body comprising a cotton matrix surrounded by a foreign body granuloma (2). Gossypiboma is a rare, but serious consequence of negligence during surgery that can have severe medical consequences, including chronic pain, infection and abscess formation (2, 3). It has an estimated incidence of 1/1000-1/10000 surgeries (2, 4).

Gossypibomas are generally reported after abdominal laparotomy; however, it can occur following any surgical procedure. An intrathoracic gossypiboma is extremely rare, most of which are associated with pulmonary surgeries (5, 6). Although almost all surgical sponges contain a radiopaque marker, usually a barium sulfate filament, the diagnosis of an intrathoracic gossypiboma can be easily missed because of its rare occurrence and low index of suspicion (5). Gossypibomas can be diagnosed over variable periods after surgery, retention over longer periods being associated with greater morbidity (7). Here we report a case of an intrathoracic gossypiboma resulting in chronic cough, eight years after coronary artery bypass surgery.

## 2. Case Presentation

**Figure 1.**
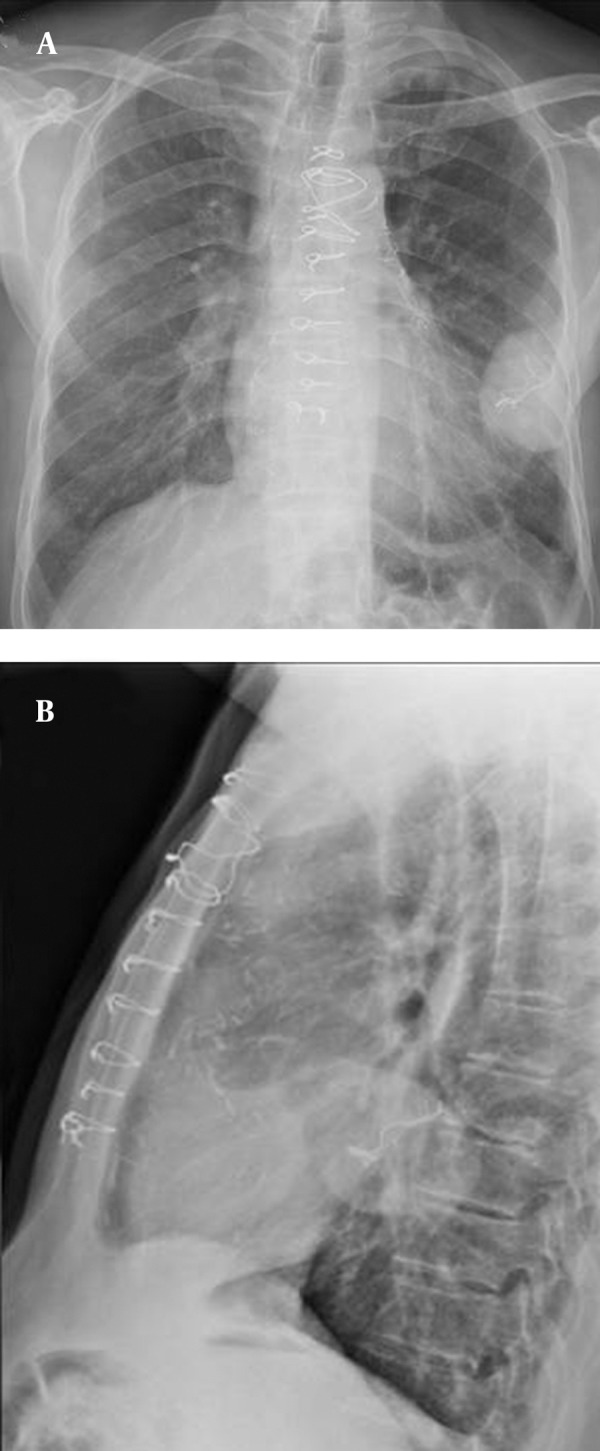
A) Posteroanterior and B) Lateral chest radiograph revealed a well-marginated homogeneous opacity in the middle-lower zone of the left hemithorax with striped appearance in the center

A 70-year-old, normotensive, nondiabetic, non-smoking male patient came to our hospital with an eight-month history of chronic recurrent cough without sputum, hemoptysis, dyspnea or fever. The symptoms were relatively resistant to inhaled corticosteroids. He had undergone coronary artery bypass surgery eight years ago. General physical examination revealed a thin male with a sternotomy scar. Physical examination of the chest revealed diminished breath sounds and fine crepitation at the left lower lung zone. Routine hematological, biochemical investigations and pulmonary function test were within normal limits. Routine chest radiographs were taken initially. The posteroanterior and lateral projection (Figure 1) revealed a well-marginated, pleural-based, homogeneous opacity at the middle-lower zone of the left hemithorax with striped appearance in the center.

**Figure 2.**
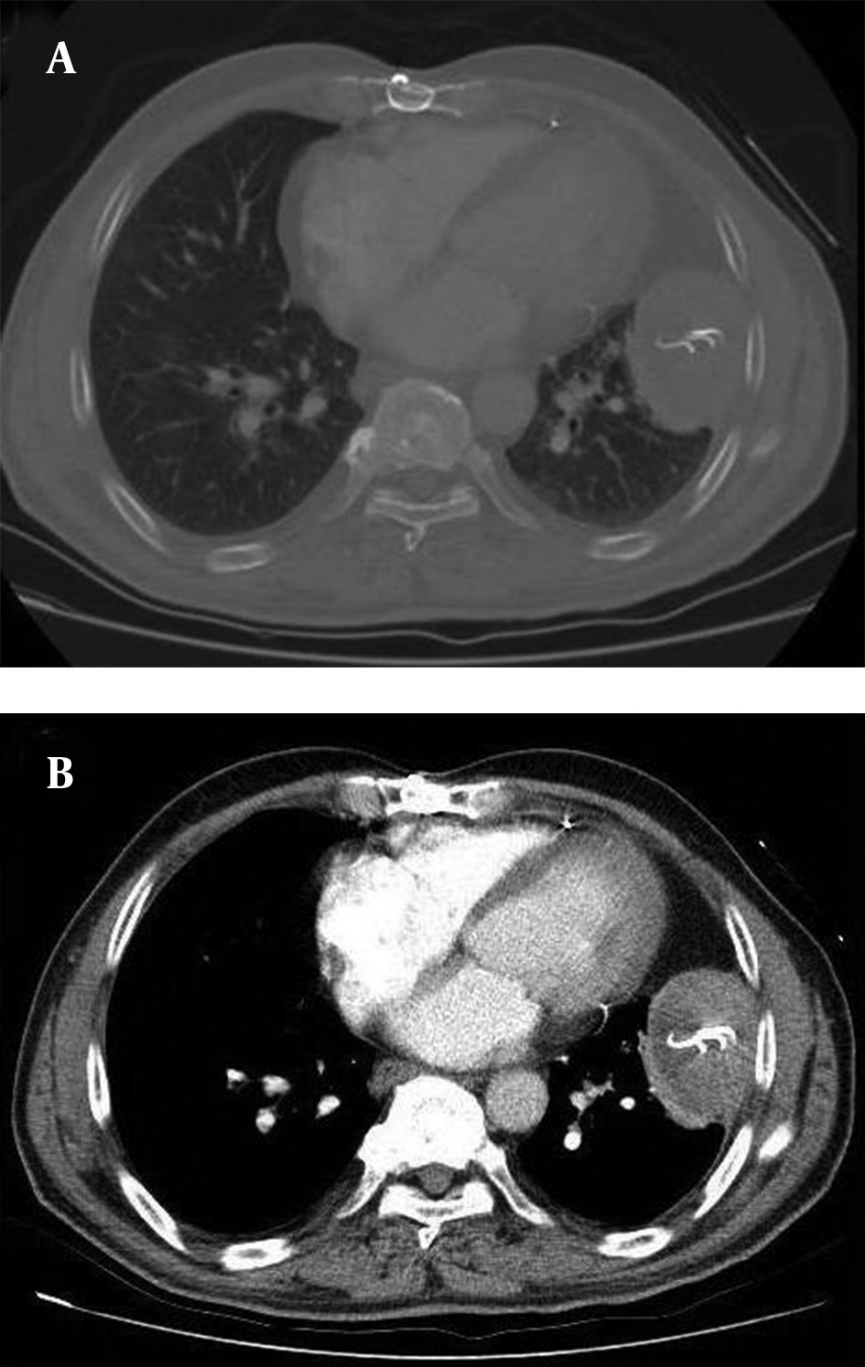
A) Parenchymal and B) Mediastinal windows of thorax CT scan revealed a pleural-based mass with a hyperdense rim capsule in the laterobasal segment of the left lower lobe with hyperdense striped appearance in the center

Thorax computed tomography (CT) scan (Figure 2) revealed a pleural-based mass with a hyperdense rim capsule, in the laterobasal segment of the left lower lobe with hyperdense rim and striped appearance in the center. Serum inflammatory markers were normal and the patient’s symptoms were not consistent with infection. We therefore did not consider it likely to be pneumonia, abcess or hydatid cyst. Although we had included benign/malignant solid mass into the differential diagnosis, radiological markers (especially the striped appearance in the center) and a history of previous thoracic surgery led to a diagnosis of an intrathoracic gossypiboma. Excisional operation was performed without diagnostic biopsy prior to the procedure.

**Figure 3.**
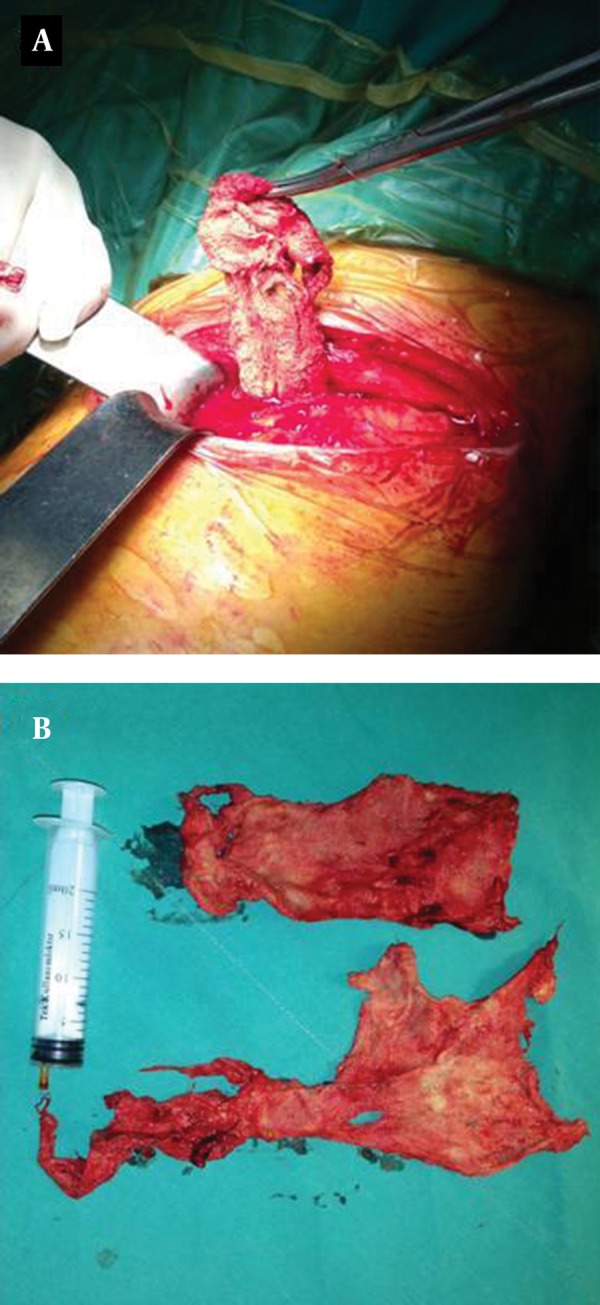
A and B) The removed mass contained strong smelling purulent material and a disintegrated surgical sponge

**Figure 4.**
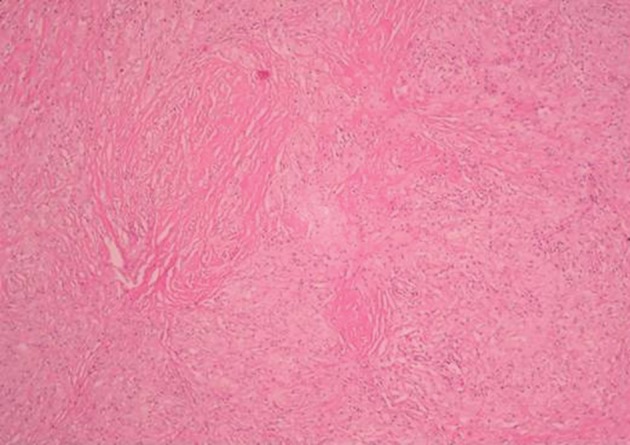
Histiocyte clusters with wide and thin dense cytoplasms in fibrous stroma (H&E, 10˟)

**Figure 5.**
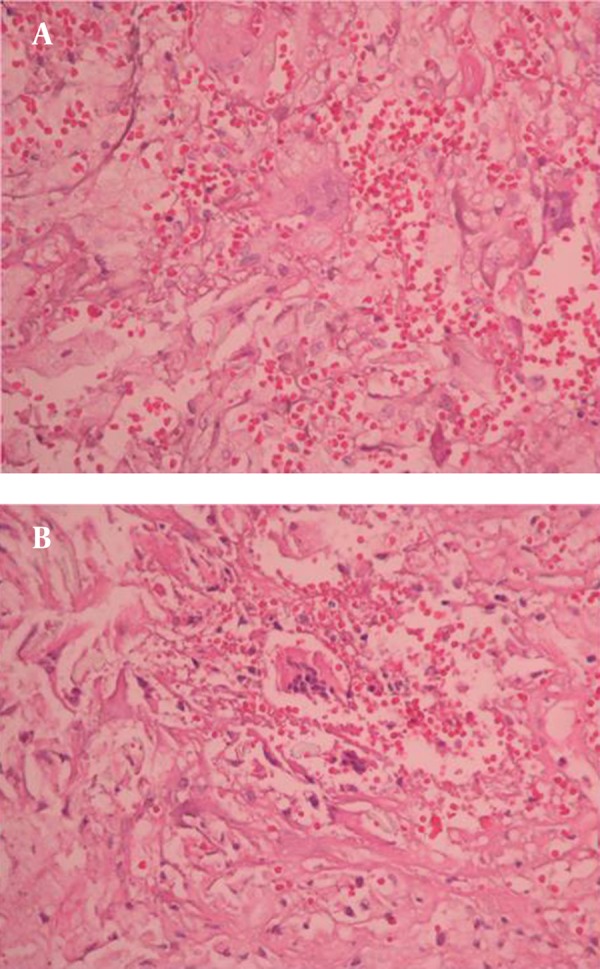
A) Foreign-body giant cells containing eccentrically placed 3-15 nuclei and fine granular cytoplasm rarely displaying demonstrable foreign material (H&E, 40˟); B) Foreign-body giant cells widely displaying demonstrable foreign material (H&E, 40˟)

An exploratory surgery of the left hemithorax was performed. On opening the thorax, dense adhesions were found. Thickening of the parietal pleura was also observed in the lower left pleural cavity. The mass contained strong-smelling purulent material and fragments of a disintegrated surgical sponge. The mass was dissected and removed (Figure 3). Additionally, the parietal pleura was decorticated. Histopathological examination revealed histiocytes distributed in clusters, with wide and thin dense cytoplasms in a fibrohyalinized stroma (Figure 4). In addition, giant multinucleated cells with 3-15 nuclei and fine granular cytoplasms containing foreign bodies were displayed (Figure 5). The patient recovered uneventfully with complete resolution of all clinical symptoms on follow-up.

## 3. Discussion

The word “gossypiboma” refers to sponges, towels or other instruments (laparotomy pads, cotton swabs and buds, drains, needles, hemostats and forceps) that are inadvertently forgotten within the human body during surgical procedures (6). It is associated with numerous complications (abscesses, fistulas, perforations, adhesions, migrations within the contiguous organs, or remobilization through the vessels) and also possible legal consequences. Although this term is frequently reported in the literature, many clinicians are unaware of this condition. Gossypibomas are probably under-reported because of the danger of public litigation associated with the condition.

Gossypibomas have most commonly been found in the abdomen (56%), pelvis (18%) and thorax (11%) with an average discovery time of 6 to 9 years after the procedure (8). Of the potential sites in the thorax where a sponge may be left, the pleural space seems to be the most likely; however, sometimes gossypibomas appear as intrapulmonary masses (9). The clinical presentation of gossypibomas may be acute or delayed. Generally many are identified and retrieved immediately or shortly after an operation, some may go unnoticed for years or even decades. Intrathoracic gossypibomas may present with fever, cough, hemoptysis and weight loss not attributable to the surgical procedure. 

Gossypibomas vary in radiological manifestations according to the location of the retained gauze, the type of foreign body reaction and the presence of a radiopaque marker. On chest radiographs, gossypibomas appear as an unusual opacity or as an atypical mass that generally does not change over time. Thoracic CT scan is the best method for detecting gossypibomas and complications, especially when a radiopaque marker is used. In the early postoperative period, CT shows a well-defined mediastinal or pleural-based soft tissue mass with a hyperdense rim, central air bubbles and a whirl-like pattern consisting of curvilinear, high density stripes. The spongiform appearance of a gossypiboma represents trapped air bubbles within the fibers of the gauze sponge in liquid media. The air trapped by the foreign material is resorbed over time, and in the absence of a radiopaque marker, lesions appear as solid masses with or without whirl-like, high-density stripes in the late postoperative period. At this stage, differentiation from other masses, such as neoplasms or degenerated hydatid cysts is difficult, even with the knowledge of the prior operation. In addition in our case we identified a hyperdense rim without characteristic air bubbles on CT scan. Signal intensity of intrathoracic gossypibomas on T1- and T2-weighted images varied among patients according to MR findings (10-14). MR imaging displayed a well-delineated formation with low signal intensity on T1-weighted images and a very high signal intensity on T2-weighted images; however, low signal intensity structures on T2-weighted images have also been reported (10-14).

In the microscopic examination of our case, there was foreign body granulomatous inflammation that was localized in the parenchyma around the gossypiboma. Foreign body granulomatous inflammation is a reaction to exogenous materials (linen or cotton materials, talc, suture materials, parasites, oil droplets, wood, metals, silica and silicon) or endogenous (hail shafts, keratin, cholesterol, urate/goutous tophi) (15). The most prominent cell in a foreign body reaction is the macrophage, additionally some granulocytes and monocytes are also present. The macrophage generally attempts to phagocyte the foreign body, but usually the material is much larger than the macrophage and is not easily degraded. Then some of the macrophages merge their cytoplasm to become multinucleated giant cells. If the foreign body is too large to be phagocytized and removed, granulation tissue, consisting of fibroblast and angioblasts in a matrix of collagen begins to form. The mass of phagocytes encapsulates the foreign body with a dense membrane of connective tissue (16). Over time, this localized lesion can undergo caseation necrosis, calcification and/or liquefaction. After fibrous encapsulation has occurred, the lesion is generally referred to as a granuloma. So a foreign body granuloma is composed of the foreign body surrounded by giant cells. In addition a zone of macrophages, lymphocytes, granulocytes around the giant cells and outmost encapsulating membrane of fibrous tissue (16).

Intrathoracic gossypibomas are rarely seen following thoracic surgeries. Patients are usually asymptomatic for a long period after the operation. The diagnosis of intrathoracic gossypiboma can easily be missed because of its rare occurrence, low index of suspicion and lack of familiarity with imaging findings. Thoracic CT is the best method for diagnosis and evaluating complications. An awareness of this condition in individuals with prior thoracic surgery and persistent respiratory symptoms should alert the physician. 
